# WNK-SPAK/OSR1 signaling pathway facilitates ictal activity via reduced neuronal chloride extrusion rate

**DOI:** 10.64898/2026.06.10.731153

**Published:** 2026-06-12

**Authors:** Volodymyr I. Dzhala, Fu Hung Shiu, Negah Rahmati, Aloe Carroll, Reagan Bae, Kristopher T. Kahle, Kevin J Staley

## Abstract

Seizures upregulate Na ^+^ -K ^+^ -2Cl ^-^ (NKCC1)-mediated Cl ^−^ influx and downregulate K ^+^ -Cl ^-^ (KCC2)-mediated Cl ^-^ efflux *via* the WNK-SPAK/OSR1 kinases, leading to cytoplasmic chloride ([Cl ^-^ ] _i_ ) accumulation, reduced GABAergic inhibition and anticonvulsant failure. Early studies found that inhibiting WNK-kinase reduced baseline [Cl ^-^ ] _i_ (E _Cl_ ) and seizures via increased KCC2 activity. However, increased KCC2 activity alone should not affect E _Cl_ whose determinants are more complex. We determined the net effects of WNK-SPAK/OSR1 pathway inhibitor WNK463 on E _Cl_ and [Cl ^-^ ] _i_ transients during spontaneous ictal-like discharges (ILDs). We found that WNK463 reduced interictal [Cl ^-^ ] _i_ but did not change baseline [Cl ^-^ ] _i_ measured in the presence of TTX. WNK463 enhanced neuronal Cl ^-^ extrusion during and after ILDs, before abolishing ILDs. Pharmacological inhibition and targeted siRNA silencing demonstrated that the anti-ictal effects of WNK463 involved both NKCC1 and KCC2. Thus, mutual NKCC1 inhibition and KCC2 activation via the WNK-SPAK/OSR1 pathway exert powerful anti-ictal effects by facilitating [Cl ^-^ ] _i_ extrusion during ILDs.

## Full Text Availability

The license terms selected by the author(s) for this preprint version do not permit archiving in PMC. The full text is available from the preprint server.

